# Simulation of human atherosclerotic femoral plaque tissue: the influence of plaque material model on numerical results

**DOI:** 10.1186/1475-925X-14-S1-S7

**Published:** 2015-01-09

**Authors:** Eoghan M Cunnane, John JE Mulvihill, Hilary E Barrett, Michael T Walsh

**Affiliations:** 1 Centre for Applied Biomedical Engineering Research (CABER), Department of Mechanical, Aeronautical, and Biomedical Engineering (MABE), Material and Surface Science Institute (MSSI), University of Limerick, Limerick, Ireland

## Abstract

**Background:**

Due to the limited number of experimental studies that mechanically characterise human atherosclerotic plaque tissue from the femoral arteries, a recent trend has emerged in current literature whereby one set of material data based on aortic plaque tissue is employed to numerically represent diseased femoral artery tissue. This study aims to generate novel vessel-appropriate material models for femoral plaque tissue and assess the influence of using material models based on experimental data generated from aortic plaque testing to represent diseased femoral arterial tissue.

**Methods:**

Novel material models based on experimental data generated from testing of atherosclerotic femoral artery tissue are developed and a computational analysis of the revascularisation of a quarter model idealised diseased femoral artery from a 90% diameter stenosis to a 10% diameter stenosis is performed using these novel material models. The simulation is also performed using material models based on experimental data obtained from aortic plaque testing in order to examine the effect of employing vessel appropriate material models versus those currently employed in literature to represent femoral plaque tissue.

**Results:**

Simulations that employ material models based on atherosclerotic aortic tissue exhibit much higher maximum principal stresses within the plaque than simulations that employ material models based on atherosclerotic femoral tissue. Specifically, employing a material model based on calcified aortic tissue, instead of one based on heavily calcified femoral tissue, to represent diseased femoral arterial vessels results in a 487 fold increase in maximum principal stress within the plaque at a depth of 0.8 mm from the lumen.

**Conclusions:**

Large differences are induced on numerical results as a consequence of employing material models based on aortic plaque, in place of material models based on femoral plaque, to represent a diseased femoral vessel. Due to these large discrepancies, future studies should seek to employ vessel-appropriate material models to simulate the response of diseased femoral tissue in order to obtain the most accurate numerical results.

## Background

The finite element (FE) method allows for the simulation of endovascular intervention through the use of a material model that is characterised by a strain energy function (SEF) to represent the highly deformable behaviour exhibited by healthy and diseased arterial tissue. The majority of FE studies develop the required material models based on *in vitro *experimental data obtained through the mechanical testing of healthy and atherosclerotic arterial tissue. However, as there are a limited number of experimental studies that mechanically characterise human atherosclerotic plaque tissue, a recent trend has emerged in current literature whereby one set of material data from a single arterial location is employed to represent the diseased tissue of numerous vascular locations as highlighted in Holzapfel et al. (2014) [1]. This trend is also true of numerical simulations of atherosclerotic femoral arteries as several studies characterise femoral plaque tissue using experimental data based on aortic plaque tissue generated by Loree et al. (1994) [2-5]. Such tissue has been shown to exhibit mechanical behaviour distinct to plaque tissue from other vascular locations [6].

This study generates material models based on experimental testing of atherosclerotic femoral artery tissue that can be employed to more accurately model diseased femoral artery tissue using computational methods. These models are generated from the mechanical behaviour of human atherosclerotic femoral plaque tissue that was characterised using uniaxial planar shear testing in Cunnane et al. (2014) [7]. Tissue samples were also biologically classified using Fourier Transform Infrared (FTIR) spectroscopy prior to mechanical testing and were classified into three groups based on increasing levels of calcified tissue content relative to lipid content to further characterise the plaque samples [7,8].

A computational analysis of the revascularisation of a highly idealised diseased femoral artery is performed using these novel material models and the effect of using these vessel appropriate material models versus material models based on atherosclerotic arterial tissue from aortic vessels generated by Loree et al. (1994) [2] is examined. This comparison of numerical results intends to examine the influence of basing the material models for numerical simulations of atherosclerotic femoral vessels on experimental data derived from atherosclerotic aortic tissue.

## Methods

### Material model

The Yeoh SEF is the material model used in this study to characterise the mechanical response of the tissue [9]. This function is a third-order reduced polynomial SEF that is suitable for characterising hyperelastic materials using uniaxial mechanical data, in tension or planar shear, as the function only depends on the first strain invariant of the Cauchy Green deformation tensor, I_1_.

(1) $$\Psi \left(I_{1}\right)=\overset{3}{\underset{\left(i=1\right)}{\sum }}C_{i0}{\left(I_{1}-3\right)}^{i}$$

C_i0 _are the material coefficients, and I_1 _is the first-strain invariant which is based on the principal stretch ratios, Eqn. 2.

(2) $$I_{1}=\lambda_{1}^{2}+\lambda_{2}^{2}+\lambda_{3}^{2}$$

The principal stretches (λ_1_, λ_2 _and λ_3_) for planar shear testing are shown in equation 3.

(3) $$\begin{array}{ccc} \lambda_{1}=\lambda , & \lambda_{2}=1, & \lambda_{3}=\frac{1}{\lambda } \end{array}$$

The Yeoh SEF is sufficient for the uniaxial test data modelled in this study as it is an isotropic model. However, anisotropic material models are recommended for arterial tissues and require that the mechanical behaviour of at least two directions (circumferential and longitudinal) be characterised. Utilising an anisotropic model such as Holzapfel and Ogden (2009) [10] also requires *in vitro *histological studies of the samples in order to determine the angle and dispersion of collagen fibers within the tissue [11-13]. As such structural information is not available regarding the data modelled in this study and the geometrical sizes of the samples only permitted uniaxial testing to failure, an isotropic model based on uniaxial testing is employed.

### Mechanical testing

Twenty femoral plaque samples were characterised, as described in [7], in order to develop the SEF necessary to model femoral plaque tissue. FTIR analysis (Spectrum 100, Perkin Elmer Inc., MA, USA, Diamond Crystal) was performed over the plaque luminal surface using the attenuated total reflectance (ATR) probe to characterise the global biological content of the samples. A background spectrum was removed and the ATR crystal was placed in direct contact with the sample. All of the spectrums were acquired using the absorbance mode with a resolution of 2 cm^-1 ^for 16 scans over the range of 4000 - 700 cm^-1^. The water spectrum was subtracted from each sample spectrum prior to peak area calculation [14]. The CH_2 _stretch peaks found between 2972 - 2845 cm^-1 ^represent the absorbance of lipid within the specimen. Also, lipid ester peaks can often be identified at 1730 cm^-1 ^and were included in the lipid peak area calculation. The calcification peak is represented by the phosphate absorbance peak in the 1180 - 900 cm^-1 ^range. The area under these peaks was measured using inbuilt software from Spectrum 100 (Perkin Elmer Inc., MA, USA). From this, the ratios of calcification to lipid (Ca:Li) present within each sample were calculated and averaged. Three distinct groups were identified from these ratios based on increasing levels of calcified tissue content relative to lipid content: lightly calcified plaques (1 < Ca:Li < 1.5), moderately calcified plaques (1.5 < Ca:Li < 2) and heavily calcified plaques (2 < Ca:Li < 3).

Samples were then subjected to uniaxial planar shear testing using a uniaxial tester and video extensometer. Samples were elongated in the circumferential direction in order to determine the mechanical response of the tissue to large deformation and the mechanically induced failure properties. Samples were preconditioned using 5 cycles to 10% stretch at a displacement rate of 0.1 mm/s and then elongated to failure at a displacement rate of 30% of gauge length per second [8]. This testing generated the experimental data necessary to characterise the femoral plaque material properties.

### Material properties

#### SFA plaque properties

**Figure 1:**
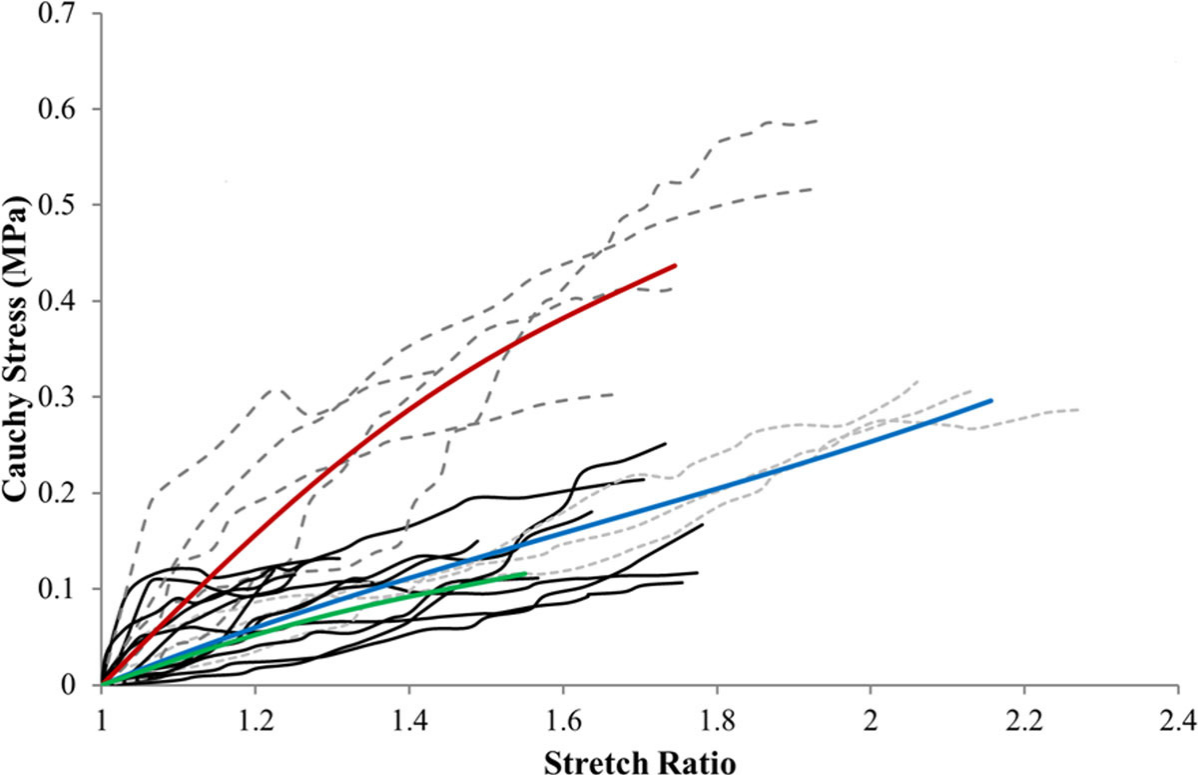
**The experimental data grouped by FTIR classification and the SEF curves fit to each data group**. The light grey dashed lines represent the lightly calcified group and the blue line characterises the average curve of this group. The dark grey dashed lines represent the moderately calcified group and the red line characterises the average curve of this group. The black lines represent the heavily calcified group and the green line characterises the average curve of this group.

The experimental data of each plaque sample were fitted to the Yeoh SEF using an optimisation technique developed with Matlab (r2010a, Natick, MA; The Mathworks Inc., 2009) to minimise the difference in stress values between the Yeoh SEF and the experimental data which ensures stability of the SEF. Figure 1 displays the experimental data, graphically represented in terms of Cauchy stress and stretch ratio, grouped by FTIR classification and also the SEF curves fit to each data group. The light grey dashed lines represent the lightly calcified group and the blue line characterises the average curve of this group which was generated by fitting a single polynomial curve to the mechanical response curves of all of the plaque samples in this group. The dark grey dashed lines represent the moderately calcified group and the red line characterises the average curve of this group. The black lines represent the heavily calcified group and the green line characterises the average curve of this group. The curves continue until the average point of mechanically induced ultimate failure recorded for each group.

The coefficients generated to characterise the Yeoh SEF curves shown in Figure 1 are displayed in table 1. These coefficients are generated from the experimental data that characterise the mechanical response of the femoral plaque tissue. The R^2 ^value denotes the quality of the fit of each set of coefficients to the corresponding average curve used to represent the experimental data.

**Table 1:** The Yeoh SEF coefficients generated to develop the material models used to represent the experimental data that characterise the mechanical response of the femoral plaque tissue.

Group	C10 (MPa)	C20 (MPa)	C30 (MPa)	R^2^
Lightly Calcified	4.98E-02	-6.19E-03	8.98E-04	0.99992
Moderately Calcified	1.35E-01	-2.84E-02	4.90E-03	0.99997
Heavily Calcified	4.62E-02	-1.47E-02	4.95E-03	0.99994

The R^2 ^values denote the quality of the fit of each set of coefficients to the corresponding average curve used to represent the experimental data.

The averaged stretch ratio and Cauchy stress values at the point of ultimate mechanically induced failure are listed in table 2. These values are used to assess the likelihood of plaque failure during revascularisation and are also compared to the failure values of aortic plaque tissue generated by Loree et al. (1994) [2].

**Table 2:** The stretch ratio and Cauchy stress values at ultimate mechanically induced failure for each of the three femoral plaque groups tested.

	Lightly Calcified		Moderately Calcified		Heavily Calcified	
Failure Type	Stretch Ratio	Cauchy (MPa)	Stretch Ratio	Cauchy (MPa)	Stretch Ratio	Cauchy (MPa)
Ultimate	2.16 ± 0.09	0.3 ± 0.01	1.75 ± 0.19	0.43 ± 0.11	1.55 ± 0.21	0.16 ± 0.04

#### Aortic plaque properties

**Figure 2:**
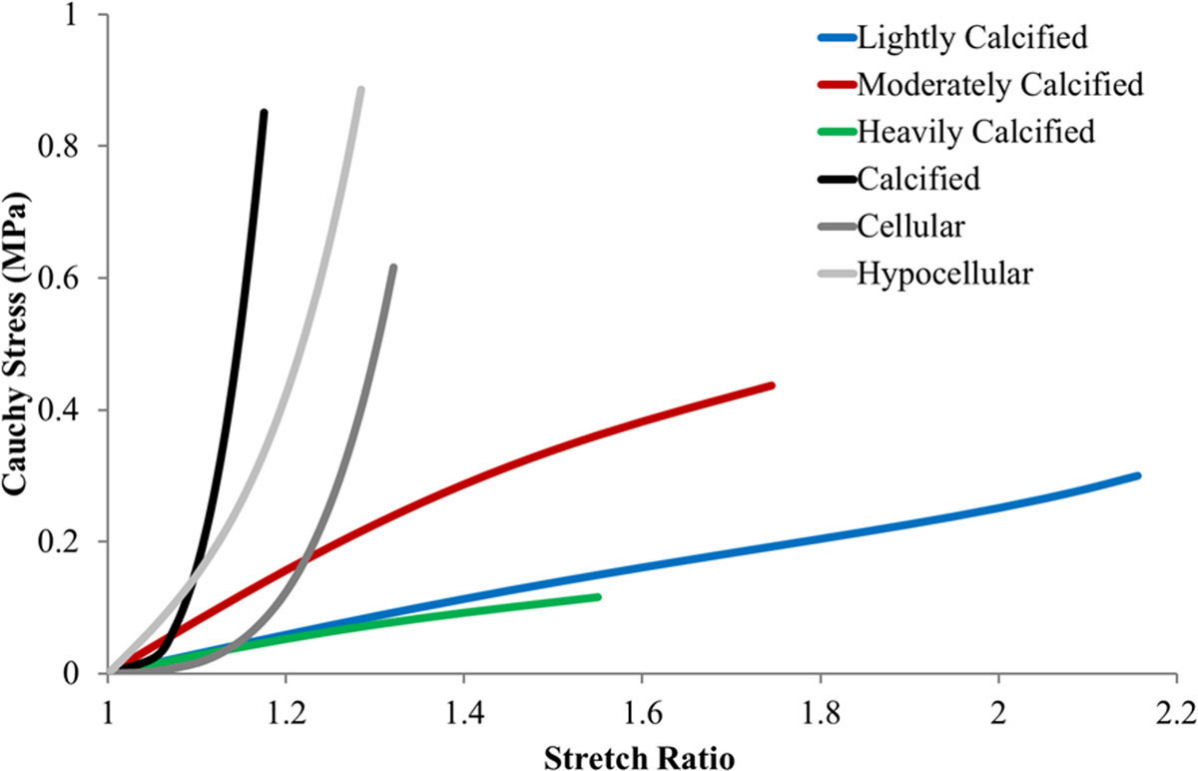
**The SEFs generated to develop the material models used to represent the atherosclerotic aortic tissue characterised by Loree et al. (1994) **[2]. Also included are the SEFs generated to develop the material models used to represent the femoral tissue. In this figure the blue, red and green lines correspond to the lightly, moderately and heavily calcified femoral groups respectively. Similarly, the black, dark grey and light grey dashed lines correspond to the calcified, cellular and hypocellular aortic groups respectively.

The aortic plaque tissue experimental data generated by Loree et al. (1994) [2] was fitted to the Yeoh SEF again using an optimisation technique developed with Matlab to minimise the difference in stress values between the Yeoh SEF and the experimental data. The data was taken from the averaged curves generated by Walsh et al. (2014) [6] which were fitted to the data published by Loree et al. (1994) [2]. This data set was selected as it is currently used in literature to computationally represent plaques from the femoral artery [4,5]. The SEFs generated to represent the atherosclerotic aortic tissue are displayed in Figure 2 and are also compared to the femoral plaque tissue material models generated specifically for this study.

Table 3 lists the SEF coefficients generated to develop the material models used to represent the material properties of the aortic plaque tissue as determined by Loree et al. (1994) [2].

**Table 3:** The Yeoh SEF coefficients generated to develop the material models used to represent the experimental data that characterise the mechanical response of aortic plaque tissue generated by Loree et al. (1994) [2].

Group	C10 (MPa)	C20 (MPa)	C30 (MPa)	R^2^
Calcified	1.41E-03	4.73E+00	8.51E-01	0.99825
Cellular	4.84E-03	3.59E-01	6.08E-01	0.99953
Hypocellular	2.20E-01	5.09E-01	6.19E-01	0.99903

The R^2 ^values denote the quality of the fit of each set of coefficients to the corresponding average curve used to represent the experimental data.

The averaged stretch ratio and Cauchy stress values at the point of ultimate mechanically induced aortic plaque tissue failure are listed in table 4. These values are included in order to determine the effect of employing plaque failure properties generated from aortic plaque tissue.

**Table 4:** The stretch ratio and Cauchy stress values at ultimate mechanically induced failure for aortic plaque tissue.

	Calcified		Cellular		Hypocellular	
Failure Type	Stretch Ratio	Cauchy (MPa)	Stretch Ratio	Cauchy (MPa)	Stretch Ratio	Cauchy (MPa)
Ultimate	1.19 ± 0.04	0.52 ± 0.34	1.42 ± 0.14	0.74 ± 0.27	1.23	0.67

### Computational model

#### Artery model

A 2D plane strain quarter model was developed to simulate an idealised concentric section of diseased femoral artery. This model is intended to offer a quantitative evaluation of the effects of employing material models based on aortic atherosclerotic tissue to represent diseased femoral artery tissue. As the numerical results of the artery model are intended solely to compare the influence of material model, geometrical and computational complexities such as patient specific geometries and compositions and also the influences of residual stresses have been neglected. A fibrous cap was not included in this model as atherosclerotic plaques originating in the femoral arteries have been found to be primarily Types VII and VIII (American Heart Association) and are therefore highly fibrotic and calcified structures that possess no fibrous cap or underlying lipid pool [15,16].

The artery dimensions and stenosis levels used to develop this model are listed in table 5. The vessel diameter used is the average value taken from several studies that characterise both the healthy and diseased femoral vessels of numerous patients using ultrasound [17-22]. The lumen diameter is the average measurement of the healthy femoral vessels of 20 patients, again obtained from a study that employs ultrasound [21]. The resulting media/adventitia wall thickness at initial stenosis is listed in table 5 and is consistent with values reported in literature for healthy femoral arteries [18]. The stenosis levels employed represent the most common scenarios found in several clinical trials that perform angioplasty procedures in the femoral arteries of multiple patients suffering from peripheral arterial disease [23-26].

**Table 5:** Dimensions and parameters used to generate the idealised diseased femoral artery modelled employed in this study.

SFA Diameter (mm)	Lumen Diameter (mm)	Wall Thickness (mm)	Initial Stenosis (% of SFA Diameter)	Final Stenosis (% of SFA Diameter)
6.87	5.95	0.48	90	10

**Figure 3:**
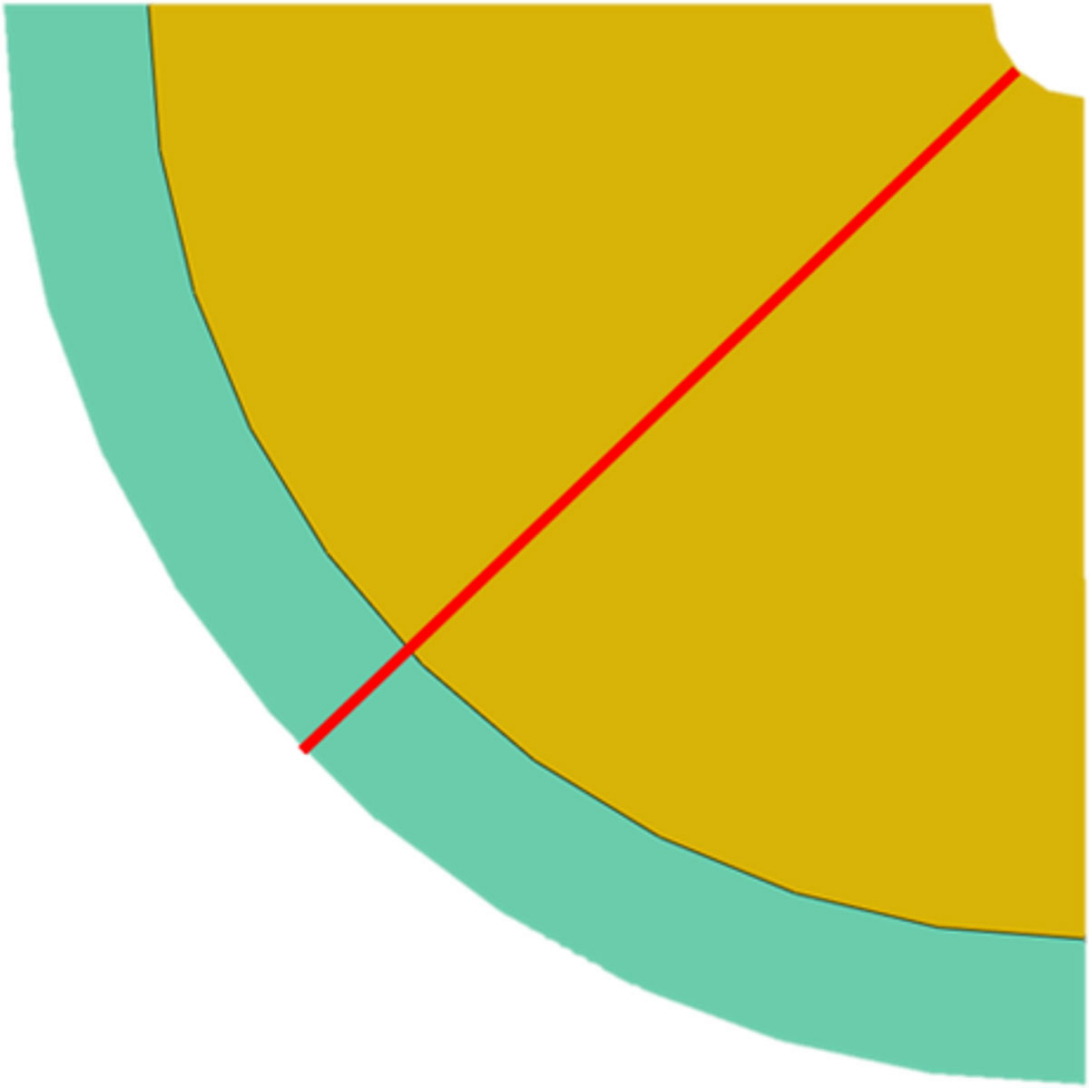
**Idealised concentric quarter model of a diseased femoral artery with an initial stenosis obstructing 90% of the luminal diameter**. The yellow section represents the diseased tissue and the green section represents the healthy tissue. The red line indicates the line along which data was extracted from the model.

A grid independent structured mesh consisting of over 400,000 4-node bilinear plane strain quadrilateral elements was generated to perform the numerical simulation. Results were obtained along a line located through the centre of the model which extends from the lumen to the extreior of the artery wall, Figure 3.

## Results

**Figure 4:**
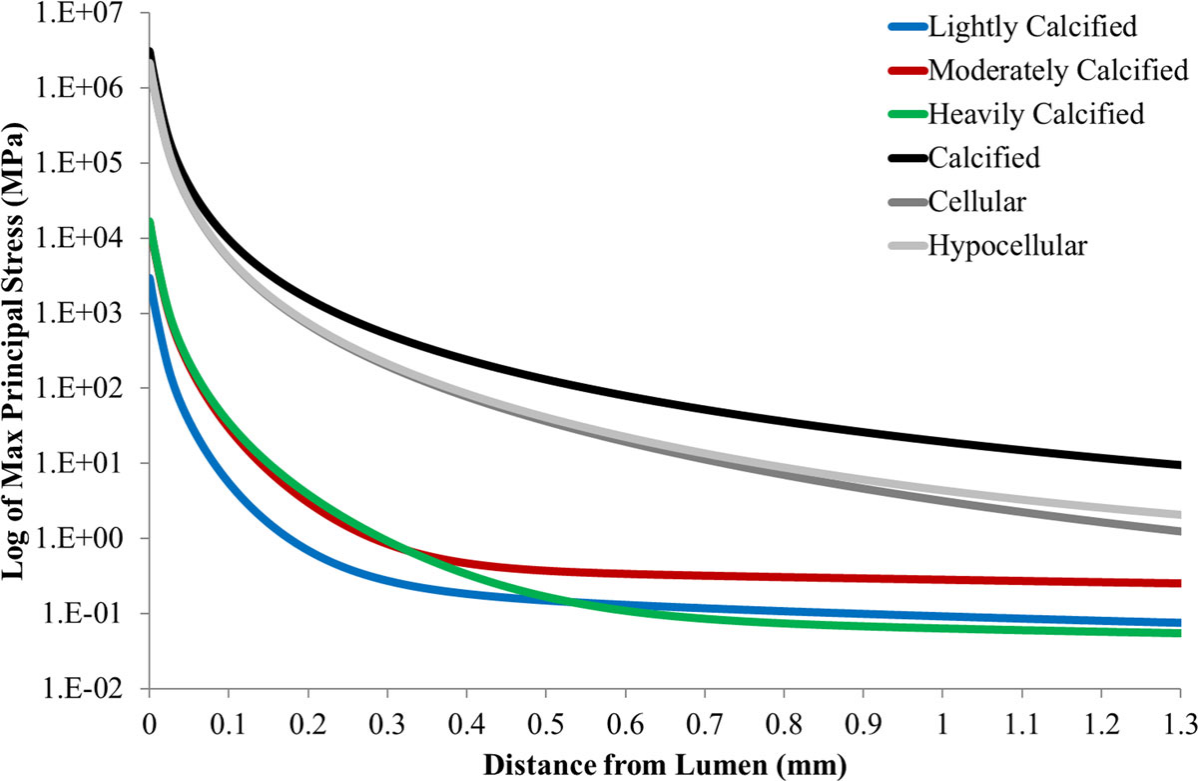
**Maximum principal stress profile across the line of interest for each material model examined in this study**. In this figure the blue, red and green lines correspond to the lightly, moderately and heavily calcified femoral groups respectively. Similarly, the black, dark grey and light grey dashed lines correspond to the calcified, cellular and hypocellular aortic groups respectively.

Figure 4 displays the maximum principal stress profiles along the line of interest, depicted in Figure 3, for each of the material models simulated in this study. A log scale is used to portray the stress values as the large differences in maximum principal stress values between the material models makes visualising the profiles difficult on a linear scale.

**Figure 5:**
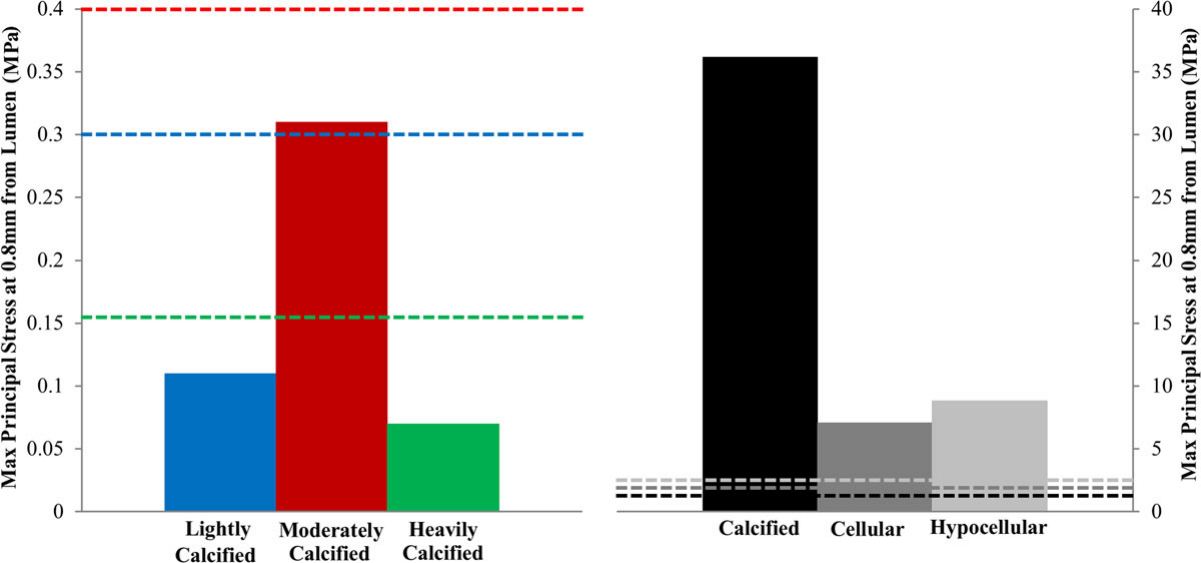
**Maximum principal stress values present in the plaque model at a distance of 0.8 mm from the lumen along the line of interest for each of the material models employed in this study**. The dashed lines represent the ultimate failure stress of each material modelled. The blue, red and green dashed lines correspond to the lightly, moderately and heavily calcified femoral groups respectively. Similarly, the black, dark grey and light grey dashed lines correspond to the calcified, cellular and hypocellular aortic groups respectively.

Figure 5 displays the individual maximum principal stress values generated by each material model at a point 0.8 mm radial from the lumen along the line of interest, Figure 3. This point was chosen to avoid the effects of the boundary conditions that were applied to the luminal surface in order to simulate the displacement of the plaque. Also, this point allows for the stress profiles to converge therefore facilitating for a better comparison of induced stress values. It can be observed that employing a material model based on calcified aortic tissue, instead of one based on heavily calcified femoral tissue, to represent diseased femoral vessels, results in a 487 fold increase in maximum principal stress at this point.

Figure 5 also illustrates the failure criteria for both the aortic and femoral data sets as listed in tables 2 and 4. These points of mechanically induced failure are portrayed as dashed lines with a colour corresponding to the material model to which it applies. These lines reveal that tissue failure would have occurred at this location of the plaque in each of the simulations that employ material models based on aortic tissue. However, tissue failure would not have occurred in the simulations that employ material models based on femoral plaque tissue.

## Discussion

This study performs a computational analysis of the revascularisation of an idealised diseased femoral artery using novel material models based on femoral plaque tissue and also using material models based on atherosclerotic aortic tissue that are currently employed in literature. The comparison of the numerical results from these simulations is intended to examine the influence of basing the material models intended for numerical simulations of atherosclerotic femoral vessels on experimental data derived from atherosclerotic aortic tissue.

The effect of employing material models based on atherosclerotic aortic tissue rather than femoral tissue can be seen in Figure 4. Comparing the results of the aortic calcified model and the heavily calcified femoral model offers the most insightful comparison as the calcified aortic experimental data is most often employed in literature to represent diseased femoral tissue [4,5] and heavily calcified samples were the most common class of samples identified by FTIR classification of atherosclerotic femoral tissue [7]. Employing a material model based on calcified aortic tissue, instead of one based on heavily calcified femoral tissue, to represent diseased femoral vessels, results in a 487 fold increase in maximum principal stress at a depth of 0.8 mm from the lumen. From a medical device design perspective, this overestimation of the stress induced in the plaque structure may lead to revascularisation devices that are inappropriately designed in order to account for this inaccurately high stress value. Rectifying this issue through the employment of vessel appropriate material models may help reduce the high incident of stent fracture [27-29] and arterial dissection [30,31] reported in literature for stents deployed in the femoral vessels.

The deviation between the numerical results of the simulations based on aortic and femoral plaque material models are caused by the discrepancies between the two experimental data sets visible in Figure 2. These discrepancies may arise due to the condition of the samples obtained from the two vascular locations. The atherosclerotic aortic samples selected for testing in the Loree et al. (1994) [2] study were visibly uncomplicated fibrous cap samples free from thrombus or surface fracture and were also separated from the underlying necrotic core. This may explain why the mechanical responses of the aortic plaque samples resemble healthy intimal tissue with an organised collagen structure [32]. A mechanical response resembling that of healthy intimal tissue is inappropriate to represent femoral plaque tissue as plaques originating in this vasculature have been shown to be highly advanced forms of atherosclerotic tissue that contain high proportions of calcified and fibrous lesions [15,16]. The femoral plaque samples are therefore believed to be far more diseased than the aortic samples and were also tested as whole specimens meaning that the mechanical responses displayed in Figure 1 incorporate the behaviour of the entire intimal layer and not just the fibrous cap. Furthermore, the majority of the mechanical responses displayed by the femoral plaque samples in Figure 1 do not display the collagen stiffening response expected of healthy arterial tissue and displayed by the aortic samples [32]. This suggests that the collagen structure is extremely heterogeneous due to the disruption caused by the advancement of the atherosclerotic process and that the plaque behaviour is dominated by the prevalence, properties and interactions of the diseased and healthy tissue components rather than properly orientated collagen fibres [33]. This potentially explains the differences between the mechanical behaviour exhibited by the two data sets and highlights the need for vessel specific plaque characterisation.

This difference in mechanical response between the two data sets is compounded further when the inappropriate aortic data is applied to numerical models that simulate the deployment of endovascular devices. Such events expose the plaque tissue to stretch that is far beyond the failure point of the tested samples. This causes the SEF curve to follow the predicted path of the tissue response and therefore any difference in mechanical behaviour, such as the stiffening behaviour of organised collagen fibres observed in the aortic plaque samples versus the heterogeneous calcified and fibrous tissue behaviour exhibited by the femoral plaque samples, is further exasperated. The problem of material model choice therefore becomes an exponential one and leads to the large discrepancies observed between the simulations based on aortic and femoral plaque material models. This highlights the need for future atherosclerotic based FE studies of femoral arteries, regardless of geometrical or computational complexity, to employ material models specific to the vascular location.

A major limitation of this study is the relative simplicity of the diseased arterial model used to compare the material models based on aortic and femoral atherosclerotic tissue. Current numerical models of diseased arterial vessels are based on 3D patient specific vessel geometries and compositions derived from medical imaging techniques [34-36]. Such models contain far more complex geometrical and computational parameters. However, as the purpose of this study is to compare the effect of material model choice on numerical results, free from geometrical influences, it was deemed acceptable to employ a highly idealised computational model. A further limitation is that the mechanical response and failure properties of atherosclerotic femoral plaque tissue are multi-axial *in vivo *parameters that *in vitro *mechanical testing cannot fully characterise. Ideally, *in vivo *imaging techniques should be employed to characterise these mechanical parameters. However, direct mechanical testing remains the only standardised method currently capable of characterising plaque failure under mechanical loading.

## Conclusion

The comparison of numerical results from simulations of femoral artery revascularisation performed in this study have revealed the influence of employing material models based on aortic and femoral atherosclerotic experimental data on numerical results. Large differences are induced on numerical results as a consequence of employing material models based on aortic plaque, in place of material models based on femoral plaque, to represent the diseased femoral vessel. These discrepancies are attributed to the differences in the condition of the aortic and femoral samples tested to generate the mechanical data whereby the aortic samples exhibit behaviour far closer to healthy intimal tissue than that of the femoral samples. Due to these large discrepancies, future studies should seek to employ vessel appropriate material models to simulate the response of diseased femoral tissue in order to obtain the most accurate numerical results.

## List of abbreviations

Ca:Li: Calcification to Lipid ratio; FE: Finite Element; FTIR: Fourier Transform Infrared; SEF: Strain Energy Function;

## Competing interests

The authors declare that they have no competing interests.

## Ethics statement

All human samples mentioned in this study were obtained from consenting patients at the University Hospital Limerick, Limerick, Ireland in a manner that conformed to the Declaration of Helsinki and was approved by the hospital's Ethical Research Committee.

## Authors' contributions

EC was the lead researcher of this paper and MW supervised all work. EC ascertained the experimental data for this study and curve-fit this data to the Yeoh function. EC also ran the finite element models for the data presented in this study. JM developed the curve-fitting code and FTIR classification method. EC, JM and MW were all involved in the conception and design of the article as well as the drafting of the manuscript. EC, JM and HB were involved in the interpretation of the data and developing the key discussion points. JM, HB and MW critically reviewed the submitted manuscript to ensure accuracy of the data and its interpretation. EC, JM, HB and MW are all accountable for every aspect of the work and ensure that the data and points made in this manuscript are accurate and are completely factual based.

## Declaration

Publication of this article was funded by the Biomedical Engineering and Regenerative Medicine (BMERM) Structured PhD programme and the framework of the Irish Government's Programme for Research in Third Level Institutions Cycle 5, with the assistance of the European Regional Development Fund.

This article has been published as part of *BioMedical Engineering OnLine *Volume 14 Supplement 1, 2015: Cardiovascular Disease and Vulnerable Plaque Biomechanics. The full contents of the supplement are available online at http://www.biomedical-engineering-online.com/supplements/14/S1
